# A New Myco-Heterotrophic Genus, *Yunorchis*, and the Molecular Phylogenetic Relationships of the Tribe Calypsoeae (Epidendroideae, Orchidaceae) Inferred from Plastid and Nuclear DNA Sequences

**DOI:** 10.1371/journal.pone.0123382

**Published:** 2015-04-22

**Authors:** Guo-Qiang Zhang, Ming-He Li, Yong-Yu Su, Li-Jun Chen, Si-Ren Lan, Zhong-Jian Liu

**Affiliations:** 1 Shenzhen Key Laboratory for Orchid Conservation and Utilization, The National Orchid Conservation Center of China and The Orchid Conservation and Research Center of Shenzhen, Shenzhen, China; 2 College of Forestry, Fujian Agriculture and Forestry University, Jinshan, Fuzhou, Fujian, China; 3 College of Forestry, South China Agricultural University, Guangzhou, China; 4 Orchid Conservation and Research Center of Fujian Agriculture and Forestry University, Jinshan, Fuzhou, Fujian, China; Saint Mary's University, CANADA

## Abstract

We identified a new holomycotrophic orchid that is related to the myco-heterotrophic Calypsoeae. Because chloroplast genes are primarily lacking or are highly divergent, key morphological characters are either reduced or lost from many myco-heterotrophs, and the phylogenetic relationships of weakly supported paraphyletic Calypsoeae within Epidendroideae have been poorly understood in previous molecular systematic studies. Using chloroplast *rbcL*, *psaB*, and *matK* and nuclear Xdh and ITS sequences, we determined the circumscription and systematic positions of the new orchid and the tribe. The results indicate that the epidendroid taxa include most of the clades that are successively sister to the grade of clades representing previously recognized tribes. Calypsoeae comprising four well-supported clades with 12 genera (except for the previous temporarily placed *Wullschlaegelia*) is supported as a monophyletic and sister clade to Epidendreae (excluding Coeliinae). The new orchid is nested in Calypsoeae and is a sister to *Dactylostalix* and/or *Calypso*. This new holomycotrophic orchid presents a subumbel inflorescence that grows underground, and flower with a long pedicel reputing the ground to open and two fragments at the base of the hook, which are obviously morphologically different from those of Calypsoeae. To accommodate this species in the current generic circumscription, a new genus *Yunorchis* was created.

## Introduction

Nonphotosynthetic mycorrhizal plants have long attracted the attention of botanists and mycologists and have been the target of unabated controversy and speculation [1]. Worldwide, there are more than 400 species (approximately 90 genera) of fully myco-heterotrophic/holomycotrophic and almost 20 000 partially myco-heterotrophic/heteromycotrophic flowering plants [2]. Myco-heterotrophic angiosperms comprise part of the Burmanniaceae, Corsiaceae, Ericaceae, Gentianaceae, Iridaceae, Orchidaceae, Petrosaviaceae, Polygalaceae, Thismiaceae, and Triuridaceae families. Among these families, Orchidaceae occupies a large number of groups [3]. The majority of holomycotrophic flowering plants are restricted to the tropics, but myco-heterotrophic Ericaceae and some Orchidaceae have been observed in temperate forests [2]. Here, we conducted a field study in the tropics of Yunnan, China, and identified a new holomycotrophic orchid that is morphologically related to the Calypsoeae tribe.

The tribe Calypsoeae (Orchidaceae, Epidendroideae) comprises approximately 12 genera and 70 species [4]. Recently, Zhai et al. [5] added one new holomycotrophic member *Danxiaorchis singchiana*. The species of the Calypsoeae tribe are typically terrestrial or myco-heterotrophs (such as *Corallorhiza*, *Danxiaorchis*, and *Yoania*) with nodes on a pseudobulb, a shrimp ridge velamen, and an occasionally lateral inflorescence with several to many flowered four pollinia in two pairs, except for *Wullschlaegelia*, which is characterized by two pollinia and was temporarily placed in Calypsoeae by Pridgeon et al. [4] based on the phylogenies of the chloroplast genes. Apart from the *Corallorhiza* (including approximately 11 species), *Govenia* (20), *Oreorchis* (16), and *Tipularia* (7), which occupy a majority of the species of this tribe, and other genera are monotypic or oligotypic, especially the saprophytic genera. Calypsoeae are distributed from Europe, northern Asia and the Americas and absent from Africa, Australia, and the islands of the East Indies and Pacific Ocean [6].

Because of the reduction or loss of key morphological characters, the taxonomic affinities of many myco-heterotrophic Calypsoeae have remained elusive for many decades [2]. The position and circumscription of the tribe Calypsoeae has been variable, and the genera that belong to this tribe have been previously included in different tribes or subtribes [7,8,9], e.g., Dressler placed *Wullschlaegelia* and *Govenia* in Gastrodiinae and Cymbidieae, respectively [8]. In addition, *Yoania australis*, a species that is endemic to New Zealand, was renamed *Danhatchia australis* and placed in Cranichideae (Orchidoideae) based on the anatomical features [4]. The molecular tools for inferring the phylogenetic position of myco-heterotrophic plants have recently become available [10]. The results of recent phylogenetic analyses of combined chloroplast genes [11,12,13,14] have provided new insight into the systematics of the tribe Calypsoeae with a paraphyly in the subfamily Epidendroideae, the first group comprising *Calypso*, *Changnienia* and *Tipularia*, and the second group comprising *Aplectrum*, *Corallorhiza*, *Cremastra*, *Danxiaorchis*, *Govenia*, and *Oreorchis*. However, the relationships between the two groups in the context of the remainder of Epidendroideae remain unclear [4].

The presence of unusual winter leaves in some of the members of the two supported groups (paraphyletic Calypsoeae within Epidendroideae) is indicative of a close relationship [4]. The results of Zhai et al. [5] suggest that Calypsoeae is a monophyletic tribe that is sister to *Gastrodia*, with a PP of 52 based on 26 genera samplings (except for 13 genera of Calypsoeae) in Epidendroideae. Although Xiang et al. [15] sampled a total of 96 genera for the phylogenetic analysis of Epidendroideae based on the combined chloroplast genes (*rbcL*, *matK* and *psaB*), these authors sampled only one supported groups (two genera, *Calypso* and *Tipularia*) of Calypsoeae, and the results indicated the two genera is sister to Dendrobieae/Malaxideae in a Bayesian inference with a PP of 0.80 and unsupported in Maximum parsimony analyses. Additionally, Pridgeon et al. [4] indicated that the two Japan endemic genera *Dactylostalix* and *Ephippianthus* form a well-supported clade within Calypsoeae based on the morphological characters. The family-wide analyses of Zhai et al. [5] indicated these two genera (without molecular data) formed a clade as sister to the *Calypso* clade based on the combined molecular markers (*rbcL*, *matK* and ITS) and morphological character matrix; however, the tribal-wide analyses formed a sister clade to the *Calypso* and *Govenia* clades. ITS was popular to permit systematic comparisons of low levels of taxa and chloroplast genes were preliminary lacking or divergent in many myco-heterotrophic Calypsoeae, both of which caused biases in inferred phylogenies [2,16]. These weak evidence and sparse sampling/molecular markers indicate that the tribal phylogenetic position within Epidendroideae and the circumscription of myco-heterotrophic Calypsoeae remain unresolved.

In this study, we used DNA sequences for the chloroplast genes *rbcL*, *psaB* and *matK* and the nuclear ITS and low-copy protein-coding gene Xdh to determine the circumscription and systematic positions of new myco-heterotrophic orchids and the Calypsoeae tribe in Epidendroideae and to infer molecular phylogenetic relationships within Calypsoeae.

## Results

### Sequence characteristics

The general features of the DNA regions that were used are presented in Table 1. The attempt to amplify *psaB* from the new myco-heterotrophic orchid (*Yunorchis pingbianensis*) failed. It is possible that this region is lacking. A total of 1047 bp of *rbcL* and 1458 bp of *matK* sequences (GenBank accession KM526763 and KM526774) were assessed, and none obvious deletion/addition loci were observed among the sequences.

**Table 1:** Parsimony statistics from phylogenetic analyses of the various datasets.

Data	Taxa	Aligned length	Information site	TL	CI	RI
**Subfamily-wide matrix**						
***rbcL***	104	1346	216 (16.05%)	1182	0.5051	0.5755
***matK***	104	1623	518 (31.92%)	2384	0.3560	0.5182
***psaB***	87	1677	197 (11.75%)	862	0.5835	0.6291
***rbcL*+ *matK*+ *psaB***	104	4646	931 (20.04%)	5140	0.4811	0.5309
**Xdh**	70	963	455 (47.25%)	2150	0.4647	0.5843
**combined**	104	5609	1386 (24.71%)	7333	0.4739	0.5438
**Tribal-wide matrix**						
***rbcL***	29	1296	113 (8.72%)	360	0.8139	0.6042
***matK***	33	1704	209 (12.27%)	497	0.8390	0.9378
***rbcL*+ *matK***	36	3000	322 (10.73%)	913	0.8160	0.8918
**ITS**	32	673	205 (30.46%)	641	0.7285	0.7573
**combined**	36	3673	527 (14.35%)	1593	0.7659	0.8362

In the subfamily-wide matrix, 104 DNA sequences of *rbcL* and *matK*, 87 of *psaB*, and 70 of Xdh were obtained. The most variable dataset was Xdh with 47.25% potentially informative sites (excluding uninformative characters). Parsimony analyses resulted in a parsimonious tree of 2150 steps (TL) with a consistency index (CI) = 0.4647 and a retention index (RI) = 0.5843. The second variable dataset was *matK* (31.92% potentially informative sites), which had the lowest CI = 0.3560 and RI = 0.5182. The third most variable dataset was *rbcL* with 16.05% potentially informative sites. The least variable dataset was *psaB* with 11.75% potentially informative sites, which had the highest CI = 0.5835 and RI = 0.6291. In the combined three chloroplast markers (*rbcL*, *matK*, *psaB*) matrix, which comprised 4646 aligned nucleotides with 931 potentially informative sites (20.04%), TL = 5140, CI = 0.4811 and RI = 0.5309. In the combined chloroplast and nuclear Xdh markers (963 bp) matrix, which comprised 5609 aligned nucleotides with 1386 potentially informative sites (24.71%), TL = 7333, CI = 0.4739 and RI = 0.5438.

In the tribe-wide matrix, 29 DNA sequences of *rbcL*, 33 of *matK*, and 32 of ITS were obtained. The most variable dataset was ITS, with 30.46% potentially informative sites. Parsimony analyses resulted in a parsimonious tree of 641 steps with the lowest CI = 0.7285 and RI = 0.7573. The second most variable dataset was *matK* with 12.27% potentially informative sites, which had the highest CI = 0.8390 and RI = 0.9378. The least variable dataset was *rbcL* with 8.72% potentially informative sites. In the combined two chloroplast markers (*rbcL*, *matK*) matrix, which comprised 3000 aligned nucleotides with 322 potentially informative sites (10.73%), TL = 913, CI = 0.8160 and RI = 0.8918. In the combined two chloroplast and nuclear ITS markers (673 bp) matrix, which comprised 3673 aligned nucleotides with 527 potentially informative sites (14.35%), TL = 1593, CI = 0.7659 and RI = 0.8362 (Table 1).

### Phylogeny of the subfamily Epidendroideae

The results from the single and combined analyses were presented by the Bayesian trees. The values of the Bayesian Posterior probabilities (PP) and Bootstrap percentage (BS_ML_ and BS_MP_) are indicated at each node. The branches that collapse in the strict consensus tree are indicated by “-”. In the nuclear Xdh analysis (S1 Fig), the results with added new molecular data are not completely in accordance with the results of Górniak et al. [11], especially the topology. The tribe Calypsoeae, including the new molecular taxa (*Dactylostalix* and *Danxiaorchis*), is supported as monophyletic with weak support (PP = 0.36, BS_ML_ = 30, and BS_MP_ = 60) and sister to Epidendreae (excluding *Coelia*).

No strongly supported, incongruent patterns of relationship were detected in the individual chloroplast markers analyses, so a combined analysis of all of the chloroplast data (*rbcL*, *matK*, and *psaB*) was performed. The trees of this analysis are shown in S2 Fig. The paraphyletic Calypsoeae (two supported groups) were observed, and the results were consistent with previous molecular studies. The nuclear and chloroplast trees that were generated by the Bayesian inference analyses (BI) were congruent with those that were retrieved by the ML and MP analyses except for the poorly supported nodes (PP < 0.90).

**Fig 1:**
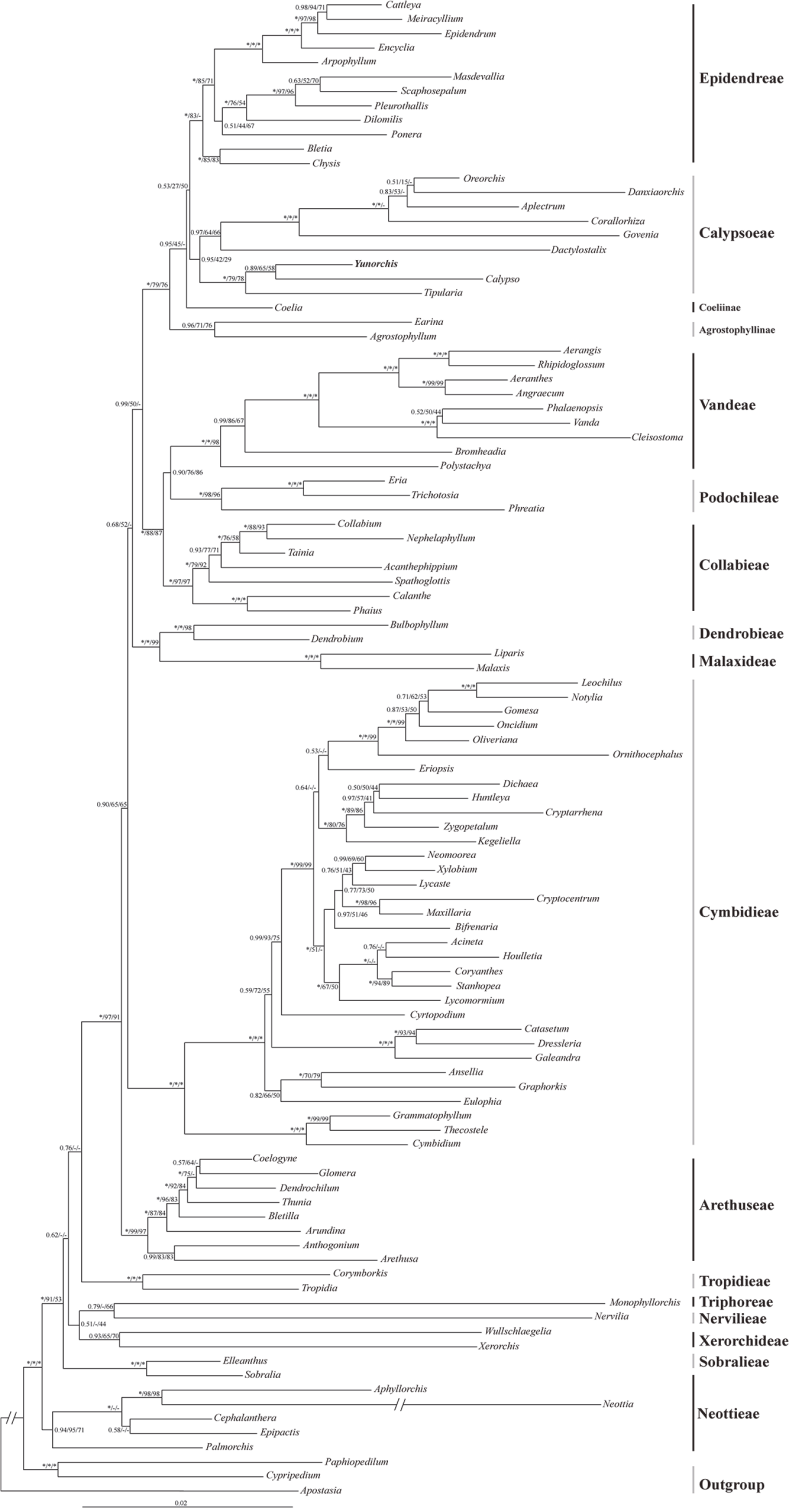
Phylogenetic relationships of the subfamily Epidendroideae based on the chloroplast DNA *rbcL*, *matK*, and *psaB* and the nuclear DNA Xdh. The numbers near the nodes are Bayesian posterior probabilities and bootstrap percentages (PP left, BS_ML_ middle, and BS_MP_ right). “*” indicates that the node is 100% supported. “-” indicates that the node is incongruent between the topology of the MP/ML trees and the Bayesian tree.

Except for Calypsoeae and/or *Wullschlaegelia*, there were no strongly supported incongruent results in the topology of the Xdh trees and the combined chloroplast trees. Although the Calypsoeae is paraphyletic in chloroplast trees, the weak support and the presence of winter leaves in some members (except for the *Wullschlaegelia*) of the two supported groups indicate a close relationship. Therefore, the chloroplast and nuclear DNA were combined into a single dataset for the phylogenetic analyses (Fig 1). Our findings are consistent with the overall topology of the trees that are produced by the BI, ML, and MP methods, except for a few collapsed nodes (such as Dendrobieae and Malaxideae). The Posterior Probability values (PP) were often higher than the Bootstrap values (BS). The tree that was recovered in the combined analysis is more similar to that based on the Xdh than the combined chloroplast.

In the Lower or “basal” Epidendroideae, highly supported clades include a monophyletic clade (PP = 1.00 and BS = 100) consisting of Neottieae sister to all of the remaining Epidendroideae, followed by Sobralieae (PP = 1.00 and BS > 50) and the other four tribes with a relatively weak bootstrap and an unstable topology. Higher Epidendroideae taxa include most of the clades that are successively (PP > 0.90 and BS > 50) sister to the grade of clades representing previously recognized tribes. Arethuseae are monophyletic as sister to all of the other Higher Epidendroideae with strong support (PP = 1.00 and BS > 90), followed by Cymbidieae (PP = 0.90 and BS = 65), and then Malaxideae plus Dendrobieae with a relatively weak bootstrap and an unstable topology. The next most supported clade (PP = 0.99) is divided into three tribes: Collabieae, Podochileae and Vandeae (excluding Agrostophyllinae) and strongly support (PP > 0.90 and BS >75) were observed between them. The last clade is divided into four groups, comprising the first group Epidendreae (excluding *Coelia*), sister to the second group Calypsoeae (excluding *Wullschlaegelia*) with a stable topology, the third group *Coelia* (PP = 0.95) with an unstable topology, and the fourth group Agrostophyllinae with strong support (PP = 1.00 and BS >75). The new holomycotrophic orchid, as a sister to *Calypso*, is a member of the tribe Calypsoeae.

### Phylogeny of Calypsoeae

Based on the ITS sequence data, the relationships in the strict consensus tree for most of the genera are resolved and well supported (PP > 0.95 and BS > 75) with only one genus (*Oreorchis*) receiving a paraphyly (S3 Fig). The basal group comprises *Calypso*, *Changnienia*, *Dactylostalix*, and *Yunorchis*. A relatively weak bootstrap in Bayesian posterior probabilities (PP = 0.61) and stable topologies in bootstrap analyses (BS = 100) between this group and others were observed. The new orchid *Yunorchis pingbianensis* is a sister to *Dactylostalix ringens* with a PP of 0.96, BS_ML_ of 64 and BS_MP_ of 85.

The 12 genera can be distinguished easily from the phylograms that are based on the chloroplast sequence data analysis. The phylogenetic topologies that are generated from BI are consistent with those from the ML and MP analyses (S4 Fig). The basal group comprises *Calypso*, *Changnienia*, *Dactylostalix*, *Tipularia*, and *Yunorchis*. *Yunorchis pingbianensis* is a sister to *Calypso bulbosa* with a PP of 0.96, BS_ML_ of 85 and BS_MP_ of 83. The chloroplast sequence data analysis showed a relatively stronger bootstrap between this group and others, with PP = 1.00 and BS > 75.

**Fig 2:**
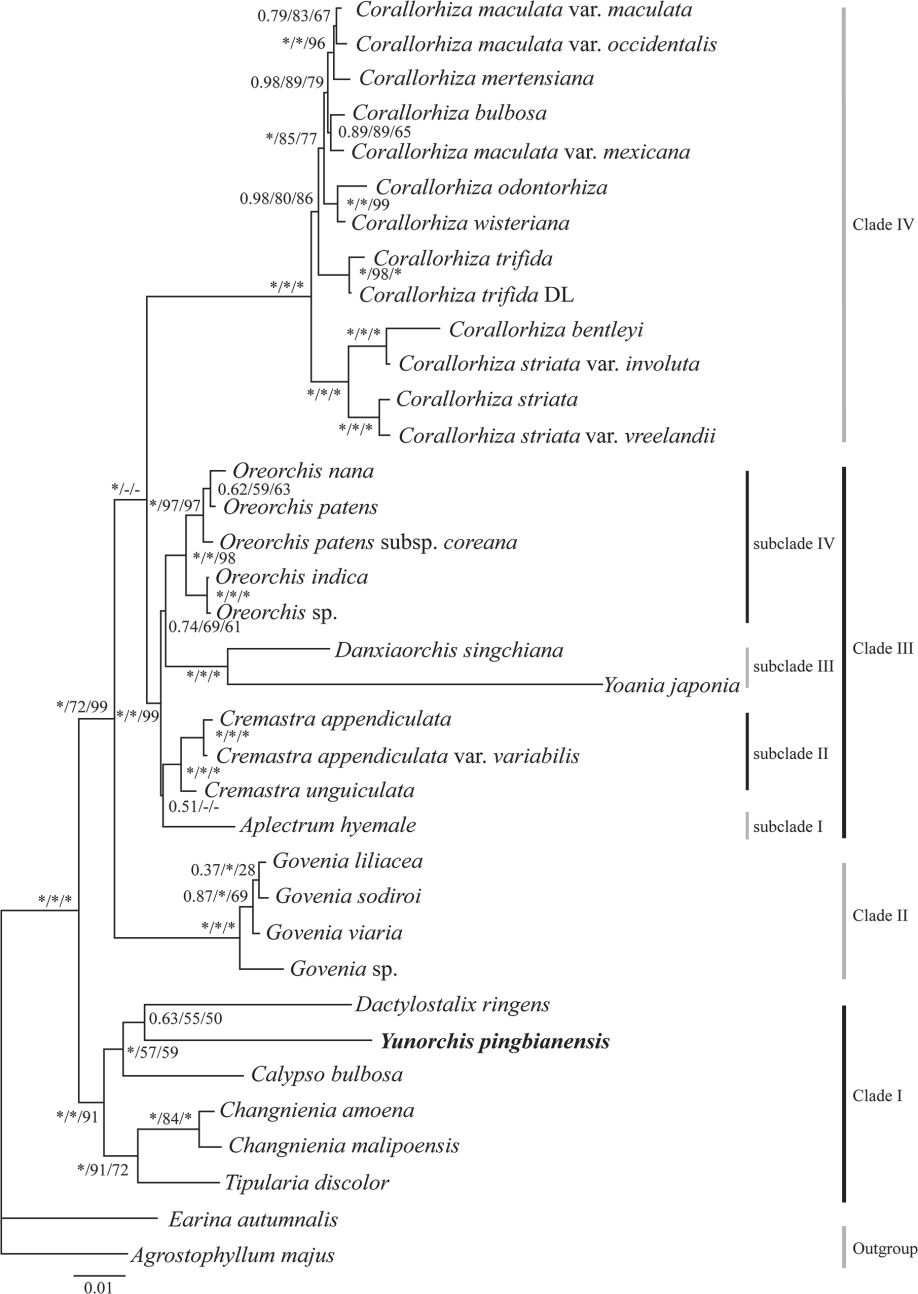
Phylogenetic relationships of the tribe Calypsoeae based on the combined nrDNA ITS and chloroplast DNA *rbcL*, *matK*. The numbers that are near the nodes are Bayesian posterior probabilities and bootstrap percentages (PP left, BS_ML_ middle, and BS_MP_ right). “*” indicates that the node is 100% supported. “-” indicates that the node is incongruent between the topology of the MP/ML trees and the Bayesian tree.

In this study, ITS, *mat*K and *rbc*L were combined into a single dataset. The results showed that the currently defined Calypsoeae is subdivided into four clades (Fig 2). Clade I, comprising the genera *Calypso*, *Changnienia*, *Dactylostalix*, *Tipularia* and *Yunorchis*, is strongly supported (PP = 1.00 and BS = 100) and is followed by the others. *Yunorchis pingbianensis*, as a sister to *Dactylostalix ringens*, has been recognized as a natural genus within this tribe. Clade II includes only the genus *Govenia*, which is strongly supported (PP = 1.00, BS_ML_ = 72, and BS_MP_ = 99) and is followed by a complex group (clade III), which includes *Aplectrum*, *Cremastra*, *Danxiaorchis*, *Oreorchis*, and *Yoania*. The topologies of this group were different, with a PP of 1.00 and a relatively weak bootstrap and an unstable topology of ML and MP phylograms (no show), and were subdivided into four subclades (Fig 2). The last clade contains a single genus, *Corallorhiza*, which comprises 13 taxa, although the phylograms showed that the data are not intragenetically well supported.

## Discussion

The chloroplast genome loci *rbcL* and *matK* have been used extensively in angiosperm phylogenetic reconstruction because of their relatively high substitution rate in combination with a conserved overall structure. Leafless, putatively achlorophyllous orchids such as *Corallorhiza* often experience deletions in their plastid genomes, and in some cases this can be severe, e.g., *Rhizanthella* (Orchidoideae), with only 59190 bp, is the least gene-rich plastid genome known to date apart from the fragmented plastid genome of some dinoflagellate [17]. Freudenstein and Doyle [18] found much less deletion in the plastid genome of *Corallorhiza* than in other leafless angiosperms that have been examined. The *rbcL* and *matK* have not been found in *Rhizanthella* [17] and a lot of deletion loci/ fragment of them were observed in *Corallorhiza* [18] and *Yoania* [5], but few loci were loss in *Danxiaorchis* and the new orchid. This gradually lost pattern seems that the leafless habit of orchids has developed several times and at least three times within Calyposeae.

### Subfamily-wide analysis of Epidendroideae

The use of multiple genes is helpful in the accuracy of phylogenetic reconstruction [19,20]. Xiang et al. [15] combined the *rbcL*, *matK*, and *psaB* sequences into a single dataset for the analysis of Epidendroideae, but the sampling and data support for Calypsoeae was weak. In this study, the data for the combined chloroplast genes (*rbcL*, *matK*, and *psaB*) indicate that the tribe Calypsoeae is divided into two supported groups (PP < 0.50, BS < 50). The results of these chloroplast analyses are consistent with those that were obtained in previous studies [11,13,14]. Górniak et al. [11] indicated that 1) the low-copy nuclear protein-coding gene of Xdh is appropriate for resolving the intrafamilial relationships, 2) the phylogenetic relationships of Xdh within Orchidaceae are consistent with those of previous analyses using other DNA data, 3) Xdh is more variable than the plastid genes *matK* and *ycf1* [21] and also can be used at the subtribal and generic levels, and 4) the “difficult” Calypsoeae taxa have been resolved better using Xdh. In this study, the results of the Xdh analyses are consistent with those of Górniak et al. [11] and those of previous studies [4,5,21], but the topologies and higher supports were not entirely consistent with the results of previous studies. The chloroplast genes in many myco-heterotrophs are primarily lacking or are highly divergent, which causes biases in inferred phylogenies [2,16]. The Calypsoeae primarily comprise holomycotrophs or heteromycotrophs that obtain carbon from a symbiotic fungus; this arrangement suggests that the chloroplast genes are lacking, deleted or highly divergent [2,22]. These results indicate that the phylogenetic systematic position of the results for Calypsoeae, which lack some chloroplast genes, would be more reasonable based on the combination of *rbcL*, *matK*, *psaB*, and Xdh into a single dataset for the phylogenetic analysis of Epidendroideae. The sister relationship of Calypsoeae and Epidendreae was also recovered in the result of Nuebig et al. [21] based on the plastid gene *ycf1* using parsimony analysis.

The relationships of *Coelia* (Coeliinae) to other genera have been problematic for more than a century. Based on multiple DNA (*rbcL*, *matK*, *trnL-F*, and ITS) analyses, van den Berg et al. [23] described a circumscription of Epidendreae that includes only five New World subtribes, namely, Bletiinae, Chysinae, Laeliinae, Pleurothallidinae, and Ponerinae, and concluded that *Coelia* is a sister to Calypsoeae. However, Pridgeon et al. [4] placed *Coelia* as a subtribe within Epidendreae based on their unpublished analyses. In our analyses (Fig 1 and S1 Fig), *Coelia* is a sister to Epidendreae plus Calypsoeae. Additionally, van den Berg et al. [23] indicated that the subtribe Agrostophyllinae of Vandeae is a sister to the “core Vandeae” (Aeridinae, Angraecinae, and Polystrachyinae) plus the tribe Cymbidieae, but the node was weakly supported. In Bayesian analysis based on the Xdh gene, Górniak et al. [11] recovered similar relationships; *Earina* (Agrostophyllinae) was far from the core Vandeae, being a sister to Epidendreae plus Calypsoeae. In our chloroplast analysis (combining *rbcL*, *matK*, and *psaB*), Agrostophyllinae was a sister to one of the groups of Calypsoeae (S2 Fig). In the nuclear Xdh (S1 Fig) and combined *rbcL*, *matK*, *psaB* and Xdh results (Fig 1), the Agrostophyllinae was a sister (PP > 0.90 and BS > 75) to the group that was formed by Coeliinae being a sister to Calypsoeae/Epidendreae. Therefore, the inclusion of Coeliinae (only *Coelia*) and Calypsoeae in Epidendreae and the system position of Agrostophyllinae warrant further study.

The systematic position of neotropical *Wullschlaegelia* has long been in trouble. Szlachetko [24] described the genus as a monotypic subtribe within Gastrodieae based on the similar seed morphology, sectile pollinia, cellular viscidium, and the lack of chlorophyll. However, these morphological data are not supported by chloroplast DNA data, suggesting that this genus nests in Calypsoeae. Based on the sequences of the chloroplast genes *rbcL* and *matK*, Pridgeon et al. [4] and Zhai et al. [5] placed this genus within Calypsoeae. Based on the sequence of nuclear Xdh, Górniak et al. [11] indicated that this genus is a sister to *Xerorchis* within the “lower” Epidendroideae. Szlachetko [24] also indicated differences in the floral morphology and the position of the anther with Gastrodieae. Because *Wullschlaegelia* is a holomycotrophic plant [25], the nuclear evidence appears to be more credible [2]. Moreover, *Wullschlaegelia* has walls in a striate-rugulate pattern with free-standing baculae between them or coarsely verrucate-striate or gemmate [4]. The pollinia of Orchidaceae have been considered informative sources of both taxonomic and phylogenetic information and are thought to be less susceptible to parallelism than other floral features [26]. Indeed, the number of pollinia in *Wullschlaegelia* is two, and the members of Calypsoeae are four. These data suggest that this genus and Calypsoeae are not closely related. Therefore, we conducted analyses with *Wullschlaegelia*, which is a sister to *Xerorchis*, based on our results (Fig 1 and S1 and S2 Figs) and the conclusion of Górniak et al. [11].

### Phylogenetic relationships within Calypsoeae

The present phylogenetic study was primarily conducted based on the molecular dataset that was described by Zhai et al. [5]. In this study, we performed an additional DNA sequence analysis using the excluded highly homoplasious morphological characters. Although the phylogenetic relationships were fully assessed and discussed in the previous study using the combined analysis of molecular and morphological characters, most of the support was weak. Based on the new molecular systematic evidence with greater support and in the interest of nomenclatural stability, we divided this tribe into four clades, and we redefined the four subtribes, namely, Aplectrinae, Cremastrinae, Wullschalaegeliinae (the genus *Oreorchis* in this study), and Yoaniinae, which were circumscribed by Zhai et al. [5], as four subclades of clade II.

In the phylogenetic analyses (Fig 2 and S3 and S4 Figs), the new holomycotrophic orchid were not nested into the same group with the other three (*Corallorhiza*, *Danxiaorchis* and *Yoania*) holomycotrophic genera of Calyposeae. The *Corallorhiza* comprising 11 species that are confined to the northern hemisphere of the New World except for one circumboreal species (*C*. *trifida*), is one of the largest leafless genera in Orchidaceae [9], and the species are characterized by the coralloid rhizome and unlobed labellum narrowing to a claw at base with two free lamellae [4]. *Danxiaorchis* is founded in Guangdong, China, and the species possesses a labellum with a large Y-shaped callus and fleshy seeds [5]. *Yoania* is sister to *Danxiaorchis* in the phylogeny of combined chloroplast *rbcL* and *matK* (Fig 2 and S4 Fig). The same relationships were observed in Zhai et al. studies [5] based on the combined molecular markers and/or morphological character matrix. These indicated that *Yoania* is most closely related to *Danxiaorchis*. Additionally, the inflorescences of *Yoania* stand straight up, the labellum bears a prominent spur and the pollinarium have four superposed pollinia [4]. However, the subumbel inflorescences of the new orchid grow underground, the sac-shaped labellum has hook and two fragments at the base of the hook, the pollinarium are in two pairs and the two pollinia of each pair are unequal in size. These features make it easy to distinguish the new orchid from the other holomycotrophic genera of Calyposeae.

The new orchid, *Yunorchis*, which is a sister to *Dactylostalix* (Fig 2 and S1 Fig) and/or *Calypso* (S2 Fig), *Changnienia*, and *Tipularia*, form an independent root clade (clade I) of Calypsoeae. The genera *Calypso*, *Dactylostalix*, and *Yunorchis* have a trilobed labellum. The lip lobe of *Calypso* occupies the first half of the sac border, the lobe base has tufted setae, and the labellum tip has two calcariform objects. This green leaf plant has an erect raceme and is primarily distributed in temperate regions. While the lip lobe of *Yunorchis* is small and is located in the middle of the labellum sac, the sac border before the lobe closes upward and forms the unciform tip. The entire plant, including the inflorescence, grows underneath the ground, and the flower stretches toward the earth’s surface when blooming; therefore, only a few flowers are observed scattering on the ground, and this plant is completely saprophytic and is distributed in tropical regions. Both genera have obvious plant morphology and habitat differences and are different from *Changnienia* and *Tipularia*, which have a distinct calcar in the labellum, green leaves, a scape growing along the ground, and inflorescences with one or many tiny flowers.

Pridgeon et al. [4] indicated that the *Dactylostalix* and *Ephippianthus* have viscidia with only the rudiments of a stipe and are unusual in having only slightly thickened rhizomes and no corms. Moreover, both genera show leaves in some seasons. Unfortunately, the molecular materials of the other genus *Ephippianthus* could not be obtained. Zhai et al. [5] indicated that the genus formed a sister with *Dactylostalix* based on morphological characteristics. The inflorescence of *Dactylostalix*, which has one or two sheathing bracts, solitary flowers, pollinarium with four superposed pollinia and a viscidium with a rudimentary stipe [4], and the few-flowered plants of *Ephippianthus*, which have membranous bracts, an entire labellum, and pollinarium with four superposed pollinia [4], are significantly different from the new holomycotrophic orchid and the other orchids of the *Calypso* Clade.

## Conclusions

The new orchid entity is restricted to Pingbian County in southern Yunnan, China, and is characterized by plants that have subumbel inflorescences growing underground. The flowers of these plants have a long pedicel rupturing the ground, a sac-shaped labellum, and a sac mouth that is connate on the front half of the edges, forming a hook that has two fragments at the base. These features distinguish the new orchid from all of the other known orchids.

Based on the combined sequences of the chloroplast genes *rbcL*, *psaB*, and *matK* and the nuclear low-copy protein-coding gene Xdh, the subfamily-wide molecular analysis revealed better topology and higher support compared to previous studies, with strong evidence that Calypsoeae is a monophyletic sister tribe to Epidendreae (except for *Coelia*) and that *Wullschlaegelia* is not a member of Calypsoeae. Based on the combined sequences of the chloroplast genes *rbcL* and *matK* and the nuclear ITS gene, the subfamily-wide molecular analysis revealed that Calypsoeae comprises four well-supported clades with 12 genera. The newly identified orchid has several distinct features, and molecular analyses indicate that this plant represents an independent lineage under the tribe Calypsoeae. This lineage should be treated as a new genus in the *Calypso* Clade with the following classification:

Subfamily: Epidendroideae

Tribe: Calypsoeae

**Fig 3:**
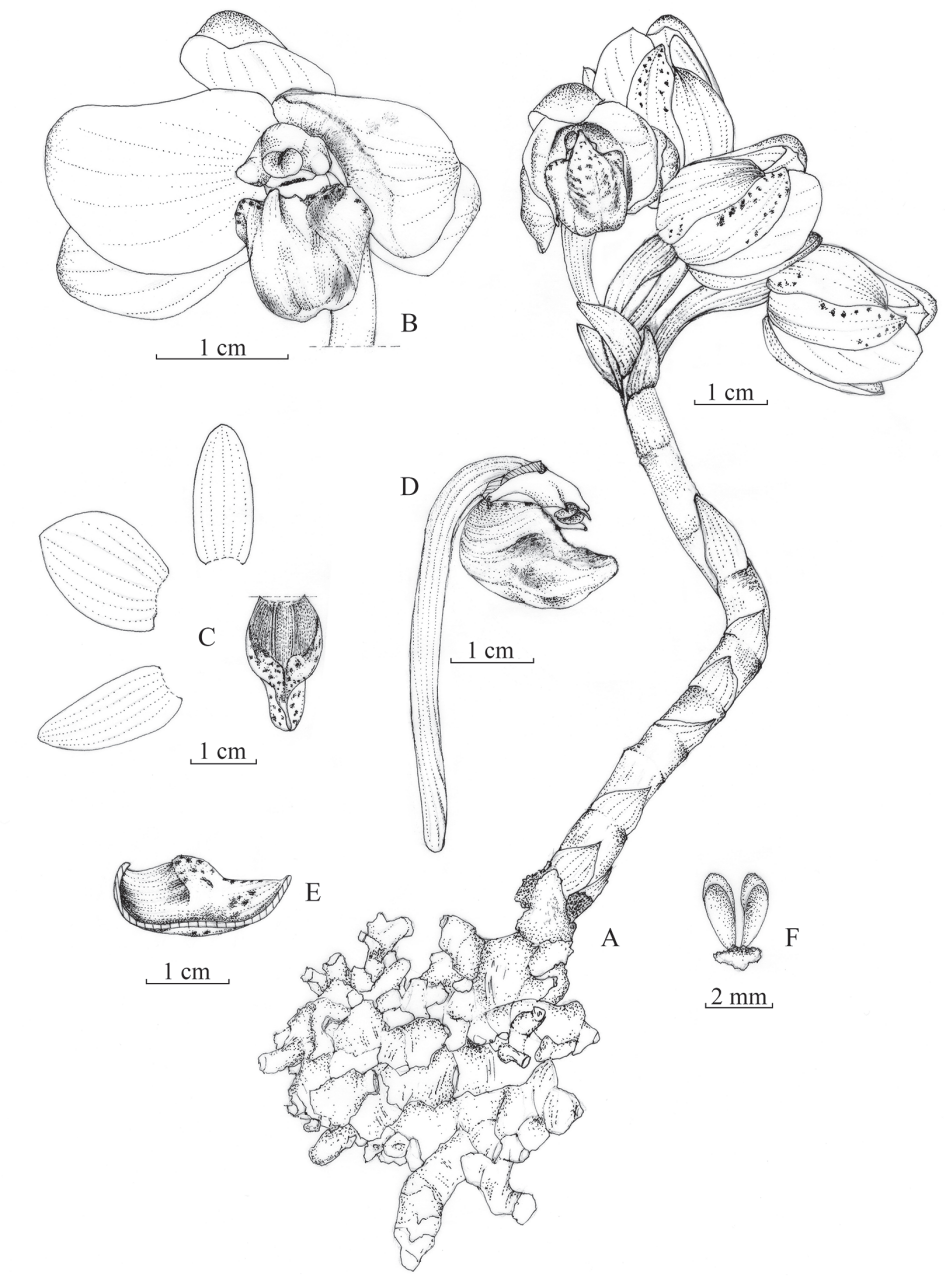
*Yunorchis pingbianensis* Z. J. Liu, G. Q. Zhang et M. H. Li: A. Flowering plant; B. Flower, front view; C. Lateral sepal, petal, dorsal sepal and lip; D. Lip and column, side view; E. Lip, longitudinal section; F. Pollinarium.

**Fig 4:**
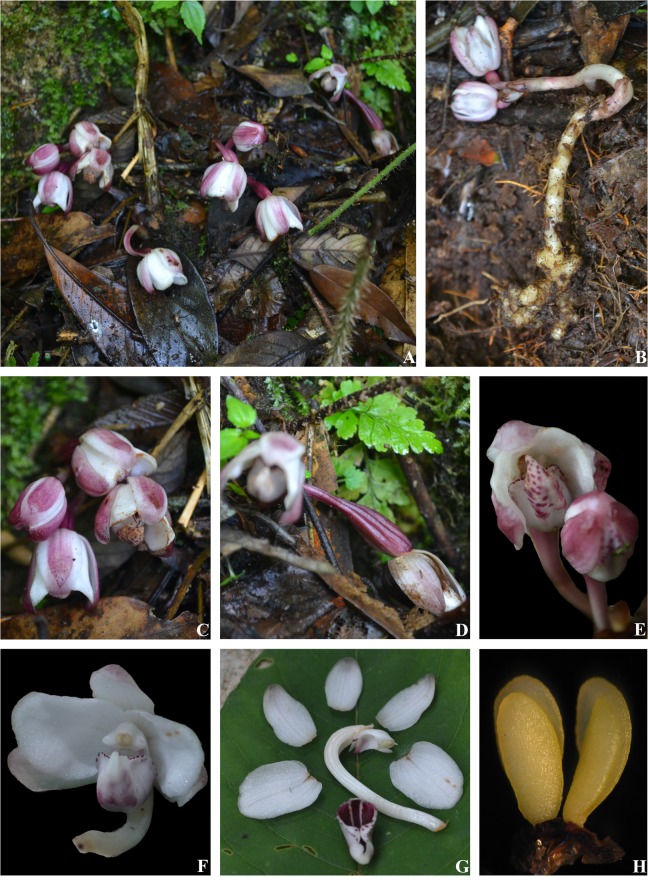
*Yunorchis pingbianensis* Z. J. Liu, G. Q. Zhang et M. H. Li: A. Flowering in nature habitat; B. Flowering plant; C. Inflorescence; D. Pedicel and ovary; E. Lip, bottom view; F. Flower, front view; G. Floral part; H. Pollinarium.

***Yunorchis pingbianensis*** Z. J. Liu, G. Q. Zhang et M. H. Li, gen. et sp. nov. (Figs 3 and 4) [urn:lsid:ipni.org:names: 77145331–1] Type: China, Yunnan, Pingbian, in a forest, alt. 2100 m, 2013. 5.31. Z. J. Liu 7103 (holotype, NOCC).

### Etymology

The generic name alludes to the type locality, Yunnan, and incorporates the Greek name for orchid; *Yunorchis* refers to an orchid genus in Yunnan province, China. The specific epithet *pingbianensis* refers to the type of species of *Yunorchis* that grows in Pingbian county of Yunnan.

### Diagnosis

This new remarkable genus is distinct from all known orchid genera; it comprises an entire plant with a subumbel inflorescence growing underground, flowers that each have a long pedicel that ruptures the ground to open and that has a sac-shaped labellum, a sac mouth that is connate on the front half edges, forming a hook and two fragments at the base of the hook, and four waxy pollinia in two pairs, each containing two pollinia that are unequal in size, and there are no conspicuous caudicles that are attached directly to a common viscidium.

### Description

Holomycotrophic plants 6–9 cm tall, rhizome tuberous, cylindrical, 4–6 cm long, 5–7 mm thick, with many short branches. Entire plant growing underground, scape arising from the terminal of rhizome, 1 or 2 inflorescences, terete, pale red-brow, 4–6-sheathed; sheaths, cylindrical, clasping scape stem, membranous, 1.0–1.5 cm long; subumbel inflorescence, 0.5–0.8 cm long, 2–6-flowered; flowers rupturing the ground when open, floral bracts broadly ovate or ovate-elliptic, 1–1.6 cm long, obtuse at apex; pedicel and ovary 3.8–5.0 cm long, glabrous, twisted; sepals pale purple-red; petals white; lip white, spotted with purple-red at apex and inside; dorsal sepals oblong, 2–2.3 cm long, 6.0–7.5 mm wide, obtuse at apex; lateral sepals ovate-elliptic, concave, with incurved margins, 2.0–2.3 cm long, 0.8–1.0 cm wide, obtuse at apex; petals narrowly oblong, 1.9–2.1 cm long, 1.6–1.8 cm wide, obtuse at apex; lip semiglobose-saccate, 1.7–1.9 cm long; sac mouth connate on front half edges forming a hook, with the lateral lobes reduced at the base of hook; disk with a purple prominent central papillate-pubescent callus extending from base to apex, column 7–8 mm long, 5.0–5.5 mm wide, with a 1.7–1.9 mm long fingerlike process projecting on either side of the anther at the apex, with a 2–3 mm long column foot at the base; anther terminal, anther cap ellipsoid, with a horn at the apex; four pollinia, in two pairs, narrowly elliptic, waxy, each pair containing two pollinia that are unequal in size, lacking conspicuous caudicles, attached directly to a common viscidium; and stigma concave, broad and large; rostellum conspicuous; capsule fusiform. Fl. May–June.

## Materials and Methods

### Ethics statement

The locations of the field studies are neither private lands nor protected areas but are controlled by the State Forestry Administration of China, with which our institution is affiliated. The State Forestry Administration authorized us to conduct scientific observations or tests in the regions that it controls. A valid permit was obtained for testing the genes of *Yunorchis*.

### Taxon and gene sampling

To reconstruct a robust phylogeny for determining the circumscription and systematic positions of Calypsoeae, we sampled as many taxa as possible from all of the tribes recognized within Epidendroideae by Chase et al. [27] and Pridgeon et al. [4,28,29]. A total of 104 genera were sampled. The outgroup included two genera from the subfamily Cypripedioideae and one genus from the subfamily Apostasioideae. The sampled strategy is not only important for examining relationships, but the inclusion of DNA sequence data from four nuclear and chloroplast regions was expected to permit possible reinterpretation of previous hypotheses that included only few of these genomes at a time [11,12,13]. A composite of slowly evolving genes was used to infer the phylogenetic relationships at the tribal/subtribal levels. All of the terminal taxa represent a single genus and include at least three of the four DNA markers (*rbcL*, *matK*, *psaB* and Xdh), except for the new orchid and *Agrostophyllum*, which include two markers (*rbcL* and *matK*). The *Dactylostalix* is sampled for the first time in a broad molecular phylogenetic study. The voucher information and GenBank accession numbers are listed in S1 Table.

A second series of analyses focused on the tribe Calypsoeae. A composite of slowly (*rbcL* and *matK*) and quickly (ITS) evolving genes and DNA regions was used to infer the phylogenetic relationships. The analysis is primarily based on the molecular dataset that was published by Zhai et al. [5] based on the combined analysis of molecular and morphological characters. In this study, we performed additional DNA extraction (six DNA sequences of *rbcL*, four of *matK*, and five of the ITS were newly obtained) excluding the highly homoplasious morphological characters for molecular phylogenetic analyses. This tribal-wide matrix included 34 in-group species and two outgroups (*Agrostophyllum majus* and *Earina autumnalis*). The voucher information and GenBank accession numbers are listed in S2 Table. DNA extraction, PCR amplification, sequencing, sequence editing and assembly were performed according to Zhang et al. [30]. The primers are showed in S3 Table.

### Phylogenetic analyses

Phylogenetic analyses were performed under Bayesian inference (BI) and the MP and ML methods. We performed the BI analysis using MrBayes 3.1.2 [31]. For the ML analysis, we used RAxML version 7.2.8 with 100 bootstrap replicates and settings as described in Stamatakis et al. [32]. The homogeneities of the subfamily Epidendroideae between nuclear Xdh and combined chloroplast DNA and the tribe Calypsoeae between nuclear ITS data and the combined chloroplast DNA dataset were tested using the incongruence length difference (ILD) test [33], as implemented in PAUP* version 4.0b10 [34]. The ILD test was conducted with 1000 replicates, each with 10 random addition sequence replicates and TBR branch swapping, maintaining no more than 100 trees per random addition replicate. Following Cunningham [35], a significance level of P = 0.01 was used for this test. The MP analyses were performed using PAUP* version 4.0b10 [34]. The test settings included 1000 replications of randomly added sequences and a heuristic search with tree bisection-reconnection branch swapping. Table 1 lists the tree length (TL), consistency index (CI), and retention index (RI).

### Nomenclature acts

The electronic version of this article in Portable Document Format (PDF) in a work with an ISSN or ISBN will represent a published work according to the International Code of Nomenclature for algae, fungi, and plants; therefore, the new names that are contained in the electronic publication of a PLoS ONE article are effectively published under that Code from the electronic edition alone. There is no longer any need to provide printed copies.

In addition, the new names that are contained in this work have been submitted to IPNI, from where they will be made and are available to the Global Names Index. The IPNI LSID 77145331–1 can be resolved and the associated information viewed through any standard web browser by appending the LSID contained in this publication to the prefix http://ipni.org/. The online version of this work is archived and available from the following digital repositories: PubMed Central, LOCKSS.

## Supporting Information

S1 Fig Phylogenetic relationships of the subfamily Epidendroideae based on the nuclear DNA Xdh. The numbers near the nodes are Bayesian posterior probabilities and bootstrap percentages (PP left, BS_ML_ middle, and BS_MP_ right). “*” indicates that the node is 100% supported. “-” indicates that the node is incongruent between the topology of the MP/ML trees and the Bayesian tree.
(TIF)

S2 Fig Phylogenetic relationships of the subfamily Epidendroideae based on the chloroplast DNA *rbcL*, *matK*, and *psaB*. The numbers near the nodes are Bayesian posterior probabilities and bootstrap percentages (PP left, BS_ML_ middle, and BS_MP_ right). “*” indicates that the node is 100% supported. “-” indicates that the node is incongruent between the topology of the MP/ML trees and the Bayesian tree.
(TIF)

S3 Fig Phylogenetic relationships of the tribe Calypsoeae based on the nrDNA ITS. The numbers near the nodes are Bayesian posterior probabilities and bootstrap percentages (PP left, BS_ML_ middle, and BS_MP_ right). “*” indicates that the node is 100% supported. “-” indicates that the node is incongruent between the topology of the MP/ML trees and the Bayesian tree.
(TIF)

S4 Fig Phylogenetic relationships of the tribe Calypsoeae based on the combined chloroplast *rbcL* and *matK*. The numbers near the nodes are Bayesian posterior probabilities and bootstrap percentages (PP left, BS_ML_ middle, and BS_MP_ right). “*” indicates that the node is 100% supported. “-” indicates that the node is incongruent between the topology of the MP/ML trees and the Bayesian tree.
(TIF)

S1 Table Taxa, voucher, and GenBank accession numbers of the Epidendroideae that were used in this study. A dash (-) indicates missing data, an asterisk (*) denotes the sequences that were obtained in this study (the voucher and location are listed in S2 Table), and the remaining sequences are from GenBank.
(DOC)

S2 Table Taxa, voucher, and GenBank accession numbers of the Calypsoeae that were used in this study. A dash (-) indicates missing data, an asterisk (*) denotes sequences that were obtained in this study, and the remaining sequences are from GenBank.
(DOC)

S3 Table Primers that were used for amplification and sequencing in this study. (DOC)

## References

[1] Bidartondo MI. The evolutionary ecology of myco-heterotrophy. New Phytologist. 2005; 167: 335–352. 15998389 10.1111/j.1469-8137.2005.01429.x

[2] Merckx V, Bidartondo MI, Hynson NA. Myco-heterotrophy: When fungi host plants. Annals of Botany. 2009; 104: 1255–1261. 10.1093/aob/mcp235 19767309 PMC2778383

[3] Leake JR. The biology of myco-heterotrophic ('saprophytic') plants. New Phytologist. 1994; 127: 171–216. 10.1111/j.1469-8137.1994.tb04272.x 33874520

[4] Pridgeon AM, Cribb PJ, Chase MC, Rasmussen FN. Genera Orchidacearum: Epidendroideae (Part one), vol. 4: 89–115. Oxford University Press, New York; 2005.

[5] Zhai JW, Zhang GQ, Chen LJ, Xiao XJ, Liu KW, Tsai WC, et al A new orchid genus, *Danxiaorchis*, and phylogenetic analysis of the tribe Calypsoeae. PLoS ONE. 2013; 8: e60371 10.1371/journal.pone.0060371 23593204 PMC3617198

[6] Stern WL, Carlsward BS. Vegetative anatomy of Calypsoeae (Orchidaceae). Lankesteriana. 2008; 8: 105–112.

[7] Chen SC, Tsi ZH, Lang KY, Zhu GH. Flora Reipublic Popularis Sinicae. 1999; 18 Science Press, Beijing.

[8] Dressler RL. Phylogeny and classification of the orchid family: Cambridge University Press; 1993.

[9] Freudenstein JV, Senyo DM. Relationships and evolution of *matK* in a group of leafless orchids (*Corallorhiza* and Corallorhizinae; Orchidaceae: Epidendroideae). American Journal of Botany. 2008; 95: 498–505. 10.3732/ajb.95.4.498 21632375

[10] Cameron KM, Chase MW, Rudall PJ. Recircumscription of the monocotyledonous family Petrosaviaceae to include *Japonolirion* . Brittonia. 2003; 55: 214–225.

[11] Górniak M, Paun O, Chase MW. Phylogenetic relationships within Orchidaceae based on a low-copy nuclear coding gene, Xdh: Congruence with organellar and nuclear ribosomal DNA results. Molecular Phylogenetics and Evolution. 2010; 56: 784–795. 10.1016/j.ympev.2010.03.003 20211743

[12] Cameron KM. Utility of plastid *psaB* gene sequences for investigating intrafamilial relationships within Orchidaceae. Molecular Phylogenetics and Evolution. 2004; 31: 1157–1180. 15120407 10.1016/j.ympev.2003.10.010

[13] Freudenstein JV, van den Berg C, Goldman DH, Kores PJ, Molvray M, Chase MW. An expanded plastid DNA phylogeny of Orchidaceae and analysis of jackknife branch support strategy. American Journal of Botany. 2004; 91: 149–157. 10.3732/ajb.91.1.149 21653371

[14] van den Berg C, Goldman DH, Freudenstein JV, Pridgeon AM, Cameron KM, Chase MW. An overview of the phylogenetic relationships within Epidendroideae inferred from multiple DNA regions and recircumscription of Epidendreae and Arethuseae (Orchidaceae). American Journal of Botany. 2005; 92: 613–624. 10.3732/ajb.92.4.613 21652439

[15] Xiang XG, Jin WT, Li DZ, Schuiteman A, Huang WC, Li JW, et al Phylogenetics of tribe Collabieae (Orchidaceae, Epidendroideae) based on four chloroplast genes with morphological appraisal. PLoS ONE. 2014; 9: e87625 10.1371/journal.pone.0087625 24498156 PMC3909211

[16] Merckx V, Chatrou LW, Lemaire B, Sainge MN, Huysmans S, Smets EF. Diversification of myco-heterotrophic angiosperms: Evidence from Burmanniaceae. BMC Evolutionary Biology. 2008; 8: 178 10.1186/1471-2148-8-178 18573195 PMC2492876

[17] Delannoy E, Fujii S, des Francs-Small CC, Brundrett M, Small I. Rampant gene loss in the underground orchid *Rhizanthella gardneri* highlights evolutionary constraints on plastid genomes. Molecular Biology and Evolution. 2011; 28: 2077–2086. 10.1093/molbev/msr028 21289370 PMC3112369

[18] Freudenstein JV, Doyle JJ. Character transformation and relationships in *Corallorhiza* (Orchidaceae: Epidendroideae) I. Plastid data. American Journal of Botany. 1994; 81: 1449–1457.

[19] Rokas A, Carroll SB. More genes or more taxa? The relative contribution of gene number and taxon number to phylogenetic accuracy. Molecular Biology and Evolution. 2005; 22: 1337–1344. 15746014 10.1093/molbev/msi121

[20] Guo YY, Luo YB, Liu ZJ, Wang XQ. Evolution and biogeography of the slipper orchids: Eocene vicariance of the conduplicate genera in the Old and New World tropics. PLoS ONE. 2012; 7: e38788 10.1371/journal.pone.0038788 22685605 PMC3369861

[21] Neubig KM, Whitten WM, Carlsward BS, Blanco MA, Endara L, Williams NH, et al Phylogenetic utility of *ycf1* in orchids: A plastid gene more variable than *matK* . Plant Systematics and Evolution. 2009; 277: 75–84.

[22] Merckx V, Bidartondo MI. Breakdown and delayed cospeciation in the arbuscular mycorrhizal mutualism. Proceedings of the Royal Society B: Biological Sciences. 2008; 275: 1029–1035. 10.1098/rspb.2007.1622 18270159 PMC2600904

[23] Carlsward BS, Whitten WM, Williams NH, Bytebier B. Molecular phylogenetics of Vandeae (Orchidaceae) and the evolution of leaflessness. American Journal of Botany. 2006; 93: 770–786. 10.3732/ajb.93.5.770 21642140

[24] Szlachetko DL. Systema orchidalium. Fragm Flor Geobot Suppl 3; 1995

[25] Martos F, Dulormne M, Pailler T, Bonfante P, Faccio A, Fournel J, et al Independent recruitment of saprotrophic fungi as mycorrhizal partners by tropical achlorophyllous orchids. New Phytologist. 2009; 184: 668–681. 10.1111/j.1469-8137.2009.02987.x 19694964

[26] Freudenstein JV, Rasmussen FN. Pollinium development and number in the Orchidaceae. American Journal of Botany. 1996; 8: 813–824.

[27] Chase MW, Cameron KM, Barrett RL, Freudenstein JV. DNA data and Orchidaceae systematics: A new phylogenetic classification In Dixon KW, Kell SP, Barrett RL, Cribb PJ, editors. Orchid conservation. Natural History Publications, Kota Kinabalu, Sabah; 2003 pp. 69–89.

[28] Pridgeon AM, Cribb PJ, Chase MC, Rasmussen FN. Genera Orchidacearum Volume 5: Epidendroideae (Part two). Oxford University Press, New York; 2009.

[29] Pridgeon AM, Cribb PJ, Chase MW, Rasmussen FN. Genera Orchidacearum Volume 6: Epidendroideae (Part three). Oxford University Press, New York; 2014.

[30] Zhang GQ, Liu KW, Chen LJ, Xiao XJ, Zhai JW, Li LQ, et al A new molecular phylogeny and a new genus, *Pendulorchis*, of the *Aerides*–*Vanda* alliance (Orchidaceae: Epidendroideae). PLoS ONE. 2013; 8: e60097 10.1371/journal.pone.0060097 23577083 PMC3618120

[31] Ronquist F, Huelsenbeck JP. MrBayes 3: Bayesian phylogenetic inference under mixed models. Bioinformatics. 2003; 19: 1572–1574. 12912839 10.1093/bioinformatics/btg180

[32] Stamatakis A, Hoover P, Rougemont J. A rapid bootstrap algorithm for the RAxML web servers. Systematic Biology. 2008; 57: 758–771. 10.1080/10635150802429642 18853362

[33] Farris JS, Källersjö M, Kluge AG, Bult C. Constructing a significance test for incongruence. Systematic Biology. 1995; 44: 570–572.

[34] Swofford DL. PAUP* Phylogenetic analysis using parsimony (* and other methods). Version 4. Sinauer Associates; 2003

[35] Cunningham CW. Can three incongruence tests predict when data should be combined? Molecular Biology and Evolution. 1997; 14: 733–740. 9214746 10.1093/oxfordjournals.molbev.a025813

