# Supplementary figures and images for: A New Myco-Heterotrophic Genus, Yunorchis, and the Molecular Phylogenetic Relationships of the Tribe Calypsoeae (Epidendroideae, Orchidaceae) Inferred from Plastid and Nuclear DNA Sequences
Source: PLoS One. 2015 Apr 22;10(4):e0123382. doi: 10.1371/journal.pone.0123382 (PMC4406536; doi:10.1371/journal.pone.0123382)

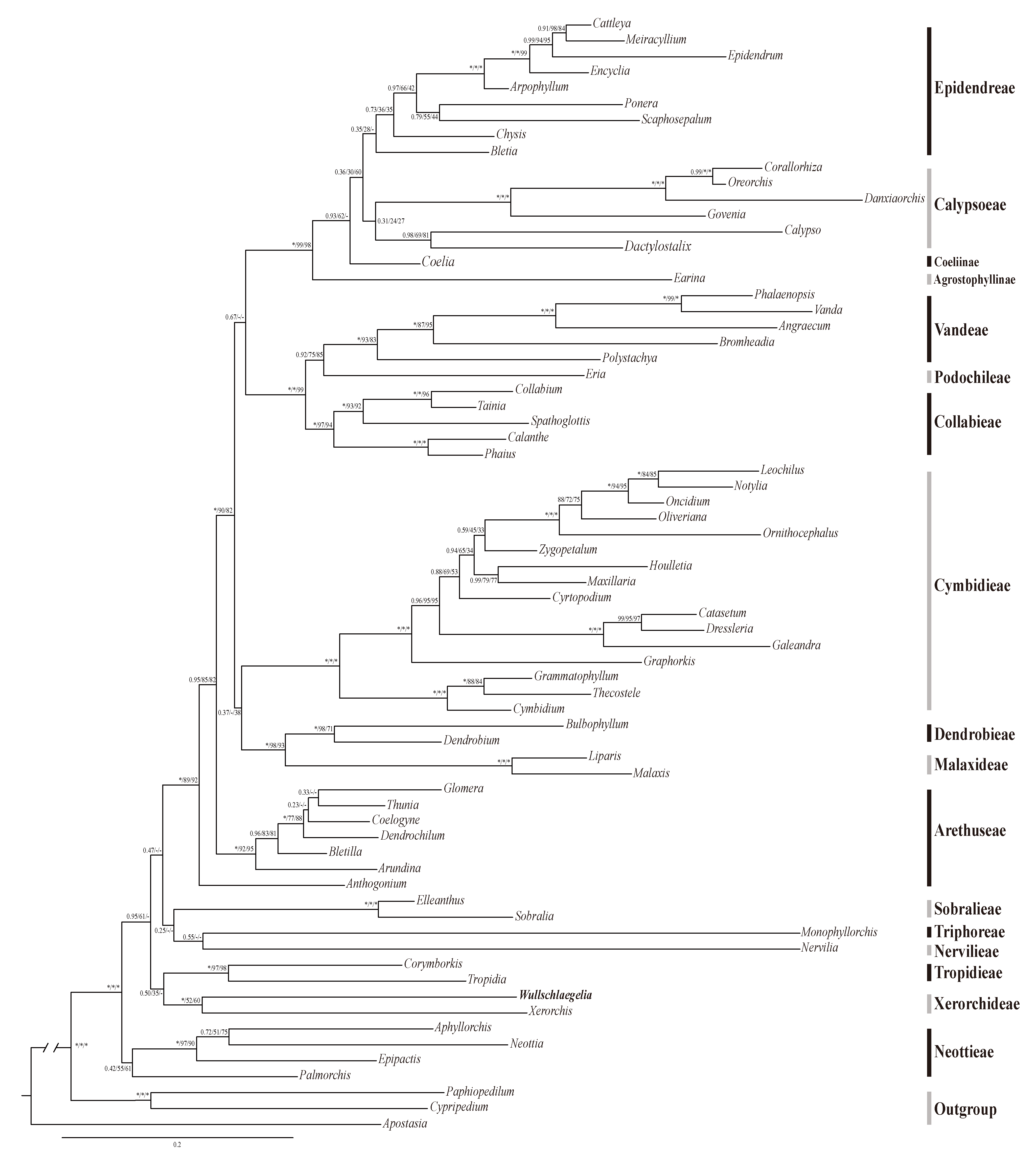

Supplement: S1 Fig — The numbers near the nodes are Bayesian posterior probabilities and bootstrap percentages (PP left, BSML middle, and BSMP right). “*” indicates that the node is 100% supported. “-” indicates that the node is incongruent between the topology of the MP/ML trees and the Bayesian tree. (TIF) [file pone.0123382.s001.tif]

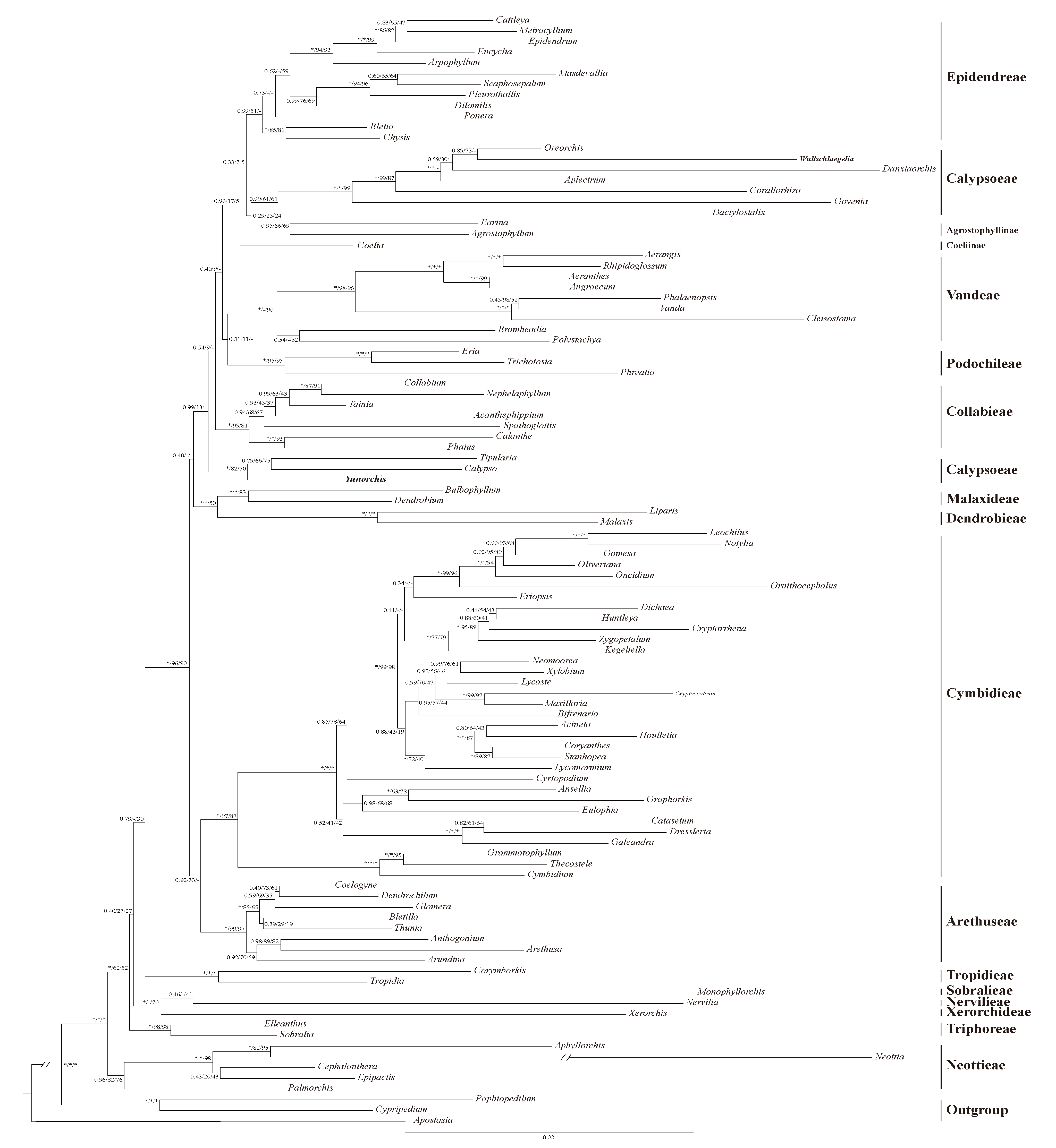

Supplement: S2 Fig — The numbers near the nodes are Bayesian posterior probabilities and bootstrap percentages (PP left, BSML middle, and BSMP right). “*” indicates that the node is 100% supported. “-” indicates that the node is incongruent between the topology of the MP/ML trees and the Bayesian tree. (TIF) [file pone.0123382.s002.tif]
