# Supplementary material for: A New Myco-Heterotrophic Genus, Yunorchis, and the Molecular Phylogenetic Relationships of the Tribe Calypsoeae (Epidendroideae, Orchidaceae) Inferred from Plastid and Nuclear DNA Sequences
Source: PLoS One. 2015 Apr 22;10(4):e0123382. doi: 10.1371/journal.pone.0123382 (PMC4406536; doi:10.1371/journal.pone.0123382)
Supplement: S1 Table — A dash (-) indicates missing data, an asterisk (*) denotes the sequences that were obtained in this study (the voucher and location are listed in S2 Table), and the remaining sequences are from GenBank. (DOC) [file pone.0123382.s005.doc]

**S1 Table.** Taxa, voucher, and GenBank accession numbers of the Epidendroideae that were used in this study. A dash (-) indicates missing data, an asterisk (*) denotes the sequences that were obtained in this study (the voucher and location are listed in Table S2), and the remaining sequences are from GenBank.

| **Genera** | **Xdh Accession**  **number** | ***rbcL* Accession**  **number** | ***psaB* Accession**  **number** | ***matK* Accession**  **number** |
| --- | --- | --- | --- | --- |
| *Acanthephippium* | - | AF074100 | AY380927 | AF263618 |
| *Acineta* | - | AF074102 | AY380929 | AF263619 |
| *Aerangis* | - | AF074103 | AY380931 | AY368389 |
| *Aeranthes* | - | AF074104 | AY380932 | AY368390 |
| *Agrostophyllum* | - | AF518054 | - | AY368391 |
| *Angraecum* | GU004415 | AF074106 | AY380933 | AF263621 |
| *Ansellia* | - | AF074107 | AF074107 | AY368392 |
| *Anthogonium* | GU004425 | AF264153 | - | AF263622 |
| *Aphyllorchis* | GU004475 | FJ454873 | - | JN706691 |
| *Aplectrum* | - | EU391356 | AY380935 | EF525693 |
| *Arethusa* | - | AF074109 | AY380938 | AF263624 |
| *Arpophyllum* | GU004454 | AF074110 | AY380939 | AF263768 |
| *Arundina* | GU004420 | AF074111 | AY380940 | EF079333 |
| *Bifrenaria* | - | AF074112 | AY380941 | DQ210752 |
| *Bletia* | GU004461 | AF264156 | AY380942 | AY121718 |
| *Bletilla* | GU004424 | AF074114 | AY380943 | EF079331 |
| *Bromheadia* | GU004402 | FN870778 | - | GQ145086 |
| *Bulbophyllum* | GU004448 | AF074115 | AY380944 | FJ794043 |
| *Calanthe* | GU004417 | AB586463 | AY380947 | HM640665 |
| *Calypso* | GU004426 | AF074120 | AY380950 | EF525689 |
| *Catasetum* | GU004392 | AF074121 | AY380951 | EF079266 |
| *Cattleya* | GU004452 | AF074122 | AY380952 | AF263816 |
| *Cephalanthera* | - | JX088502 | AY380953 | JF972943 |
| *Chysis* | GU004457 | AF074126 | AY380956 | EF079351 |
| *Cleisostoma* | - | AF074130 | AY380963 | AB217721 |
| *Coelia* | GU004458 | AF074132 | AY380966 | EF079353 |
| *Coelogyne* | GU004433 | AF074133 | - | AF302717 |
| *Collabium* | GU004404 | AF264163 | - | AF263645 |
| *Corallorhiza* | GU004436 | EU391387 | JX087681 | EF525703 |
| *Coryanthes* | - | AF074134 | AY380969 | AY368398 |
| *Corymborkis* | GU004478 | AF074136 | AY380972 | AY557203 |
| *Cryptarrhena* | - | AF074138 | AY380974 | AY368399 |
| *Cryptocentrum* | - | AF074139 | AY380975 | DQ210820 |
| *Cymbidium* | GU004389 | AF074141 | AY380978 | JX202671 |
| *Cyrtopodium* | GU004395 | AF074143 | AY380981 | AF263650 |
| *Dactylostalix* | KM526769* | KM526772* | KM526760* | KM526761* |
| *Danxiaorchis* | KM526770* | JX293187 | - | JX293186 |
| *Dendrobium* | GU004440 | KC559781 | AY380983 | KF143705 |
| *Dendrochilum* | GU004422 | AF264164 | AY380984 | AY003874 |
| *Dichaea* | - | AF074149 | AY380986 | EU123657 |
| *Dilomilis* | - | AF074150 | AY380987 | AF263765 |
| *Dressleria* | GU004391 | AF074153 | AY380991 | EF079265 |
| *Earina* | GU004428 | AF074155 | Y380993 | AY121741 |
| *Elleanthus* | GU004463 | AF074156 | AY380994 | EF079359 |
| *Encyclia* | GU004453 | AF074157 | AY380995 | AY396113 |
| *Epidendrum* | GU004451 | AF518060 | AY380996 | AF263779 |
| *Epipactis* | GU004476 | JX094817 | AY380998 | AF263659 |
| *Eria* | GU004421 | AF074164 | AY381004 | AF263660 |
| *Eriopsis* | - | AF074167 | AY381007 | DQ461806 |
| *Eulophia* | - | AF074170 | AY381010 | EF079257 |
| *Galeandra* | GU004393 | AF074171 | AY381011 | AY368408 |
| *Glomera* | GU004438 | AF074172 | AY381013 | AY121742 |
| *Gomesa* | - | FJ534252 | FJ534373 | FJ565029 |
| *Govenia* | GU004437 | AF074175 | AY381017 | EF525690 |
| *Grammatophyllum* | GU004399 | AF074176 | AY381018 | EF079262 |
| *Graphorkis* | GU004390 | FN870831 | - | AY368410 |
| *Houlletia* | GU004397 | AF074178 | AY381020 | AF239467 |
| *Huntleya* | - | AF074179 | AY381021 | EU123674 |
| *Kegeliella* | - | AF074181 | AY381024 | AF239460 |
| *Leochilus* | GU004379 | FJ534194 | FJ534316 | FJ564936 |
| *Liparis* | GU004450 | AF074183 | AY381026 | AF263667 |
| *Lycaste* | - | AF074185 | AY381028 | AF239438 |
| *Lycomormium* | - | AF074186 | AY381029 | AY368414 |
| *Malaxis* | GU004449 | AF074188 | AY381031 | AB290898 |
| *Masdevallia* | - | AF074189 | AY381032 | AF265445 |
| *Maxillaria* | GU004384 | AF074190 | AY381033 | DQ210898 |
| *Meiracyllium* | GU004462 | AF074192 | AY381037 | AF263767 |
| *Monophyllorchis* | GU004470 | AF074195 | AY381040 | EF065603 |
| *Neomoorea* | - | AF074198 | AY381042 | DQ210743 |
| *Neottia* | GU004472 | JF325876 | - | EF079303 |
| *Nephelaphyllum* | - | AF264170 | AY381043 | AF263674 |
| *Nervilia* | GU004465 | AF074199 | AY381044 | HQ848211 |
| *Notylia* | GU004380 | FJ534208 | FJ534330 | FJ564961 |
| *Oliveriana* | GU004385 | FJ534174 | FJ534296 | EF079202 |
| *Oncidium* | GU004378 | AF074201 | AY381046 | FJ564795 |
| *Oreorchis* | GU004435 | JQ933425 | - | EU266418 |
| *Ornithocephalus* | GU004381 | FJ534233 | FJ534355 | FJ565083 |
| *Palmorchis* | GU004471 | AF074206 | AY381051 | AJ310052 |
| *Phaius* | GU004432 | AF074210 | AY381053 | EF079306 |
| *Phalaenopsis* | GU004411 | AY916449 | AY381054 | AB217750 |
| *Phreatia* | - | AF074214 | AY381056 | AY368425 |
| *Pleurothallis* | - | AF518042 | AY381059 | AF302646 |
| *Polystachya* | GU004406 | AF074222 | AY381064 | AY368426 |
| *Ponera* | GU004460 | AY368368 | - | AF263764 |
| *Rhipidoglossum* | - | AF074147 | AY380985 | EF065573 |
| *Scaphosepalum* | GU004455 | AF518041 | - | AF265458 |
| *Sobralia* | GU004464 | AF074228 | AY381076 | AF263681 |
| *Spathoglottis* | GU004431 | AF264175 | AY381077 | AJ310071 |
| *Stanhopea* | - | AF074230 | AY381079 | AY368430 |
| *Tainia* | GU004410 | AF264176 | - | EF079342 |
| *Thecostele* | GU004401 | AY368371 | - | AY368431 |
| *Thunia* | GU004439 | AF074233 | AY381083 | AF302706 |
| *Tipularia* | - | AF074234 | AY381084 | AF263685 |
| *Trichotosia* | - | AF074235 | AY381085 | AY368432 |
| *Tropidia* | GU004477 | AF074237 | AY381087 | AF263686 |
| *Vanda* | GU004413 | DQ195036 | - | AB217772 |
| *Wullschlaegelia* | GU004466 | AY368436 | - | AY368434 |
| *Xerorchis* | GU004468 | AF074244 | AY381096 | AF263688 |
| *Xylobium* | - | AF074245 | AY381097 | DQ210692 |
| *Yunorchis* | - | KM526774* | - | KM526763* |
| *Zygopetalum* | GU004387 | AF074246 | AY381098 | EU123676 |
| *Apostasia* | GU004525 | HQ182416 | AY380937 | JX903642 |
| *Cypripedium* | GU004508 | AF074142 | AY380980 | AY557208 |
| *Paphiopedilum* | GU004516 | AF074209 | AY381052 | AY368379 |
