# Supplementary material for: A New Myco-Heterotrophic Genus, Yunorchis, and the Molecular Phylogenetic Relationships of the Tribe Calypsoeae (Epidendroideae, Orchidaceae) Inferred from Plastid and Nuclear DNA Sequences
Source: PLoS One. 2015 Apr 22;10(4):e0123382. doi: 10.1371/journal.pone.0123382 (PMC4406536; doi:10.1371/journal.pone.0123382)
Supplement: S2 Table — A dash (-) indicates missing data, an asterisk (*) denotes sequences that were obtained in this study, and the remaining sequences are from GenBank. (DOC) [file pone.0123382.s006.doc]

**S2 Table.** Taxa, voucher, and GenBank accession numbers of the Calypsoeae that were used in this study. A dash (-) indicates missing data, an asterisk (*) denotes sequences that were obtained in this study, and the remaining sequences are from GenBank.

| **Species** | **Voucher** | **Location** | **ITS Accession**  **number** | ***matK* Accession**  **number** | ***rbcL* Accession**  **number** |
| --- | --- | --- | --- | --- | --- |
| *Aplectrum hyemale* |  |  | EU266404 | EU266416 | FJ445516 |
| *Calypso bulbosa* |  |  | AF521076 | EF525689 | AF264162 |
| *Changnienia malipoensis* |  |  | JX293179 | JX293183 | JX293188 |
| *C. amoena* | *Z. J. Liu 52282* | Yunnan, China | KM873667* | KM873668* | KM873666* |
| *Corallorhiza bentleyi* |  |  | JF319668 | EF525706 | JF319769 |
| *C. bulbosa* |  |  | EU391332 | EF525699 | EU391366 |
| *C. maculata* var. *maculata* |  |  | EU391329 | EF525700 | EU391363 |
| *C. maculata* var. *mexicana* |  |  | EU391331 | - | EU391365 |
| *C. maculata* var. *occidentalis* |  |  | EU391330 | EF525697 | EU391364 |
| *C. mertensiana* |  |  | EU391333 | EF525704 | EU391367 |
| *C. odontorhiza* |  |  | EU391326 | EF525701 | EU391359 |
| *C. striata* |  |  | EU391349 | EF525702 | FJ445537 |
| *C. striata* var. *involuta* |  |  | EU391347 | EF525698 | FJ445565 |
| *C. striata* var. *vreelandii* |  |  | EU391352 | EF525705 | GU223951 |
| *C. trifida* DL | *Z. J. Liu 5504* | Sichuan, China | JX293181 | - | KM526771* |
| *C. trifida* |  |  | EU391324 | EF525695 | EU391357 |
| *C. wisteriana* |  |  | EU391327 | EF525703 | EU391361 |
| *Cremastra appendiculata* | *Z. J. Liu 3971* | Yunnan, China | KM526764* | JX293182 | JX293189 |
| *C. appendiculata* var. *variabilis* |  |  | EU266414 | EU266421 | - |
| *C. unguiculata* |  |  | EU266415 | EF525692 | - |
| *Danxiaorchis singchiana* | *Z. J. Liu 6038* | Guangdong, China | JX293178 | JX293186 | JX293187 |
| *Dactylostalix ringens* | *Z. J. Liu 8025* | Yunnan, China | KM526765* | KM526761* | KM526772* |
| *Govenia liliacea* |  |  | AF521056 | AY121723 | - |
| *G. sodiroi* |  |  | - | EU490695 | - |
| *G.* sp. |  |  | EF525672 | EF525690 | AF074175 |
| *G. viaria* |  |  | - | EU214357 | - |
| *Oreorchis indica* |  |  | JX293180 | JX293184 | JX293191 |
| *O. nana* | *Z. J. Liu 5509* | Sichuan, China | KM526766* | JX293185 | JX293192 |
| *O. patens* | *Z. J. Liu 4551* | Yunnan, China | KM526767* | KM526762* | KM526773* |
| *O. patens* subsp. *coreana* |  |  | EU266410 | EU266417 | - |
| *O.* sp. |  |  | EU266413 | EU266420 | - |
| *Tipularia discolor* |  |  | - | AF263685 | AF074234 |
| *Yoania japonica* | *Z. J. Liu 6241* | Fujian, China | - | - | JX293190 |
| *Yunorchis pingbianensis* | *Z. J. Liu 7103* | Yunnan, China | KM526768* | KM526763* | KM526774* |
| *Agrostophyllum majus* |  |  | AF521080 | AY368391 | AF518054 |
| *Earina autumnalis* |  |  | AY008469 | AF263656 | AF074155 |
