# Supplementary material for: A New Myco-Heterotrophic Genus, Yunorchis, and the Molecular Phylogenetic Relationships of the Tribe Calypsoeae (Epidendroideae, Orchidaceae) Inferred from Plastid and Nuclear DNA Sequences
Source: PLoS One. 2015 Apr 22;10(4):e0123382. doi: 10.1371/journal.pone.0123382 (PMC4406536; doi:10.1371/journal.pone.0123382)
Supplement: S3 Table — (DOC) [file pone.0123382.s007.doc]

**S3 Table**. Primers that were used for amplification and sequencing in this study.

| **Primer** | **Sequence（5’→3’）** | **Origin** |
| --- | --- | --- |
| Xdh-646F | TAACTCGGTGTACACTAAATCAT | This study |
| Xdh-1287R | GAAATTGGACTAGCAGTGCAGAT | This study |
| ITS A | GGAAGGAGAAGTCGTAACAAGG | Mike T, et al. [1] |
| ITS B | CTTTTCCTCCGCTTATTGATATG | Mike T, et al. [1] |
| *rbcL*-1F | ATGTCACCACAAACAGAAAC | Sulaiman SF, et al. [2] |
| *rbcL*-516F | TGTACTATTAAACCAAAATTGGG | Zhai JW, et al. [3] |
| *rbcL*-880F | CAGGCTGGTACAGTAGTGGG | Zhai JW, et al. [3] |
| *rbcL*-1285F | GCATGTGTACAAGCTCGTAATGAG | Zhai JW, et al. [3] |
| *rbcL*-1360R | CTTCACAAGCAGCAGCTAGTTC | Reeves G, et al. [4] |
| *rbcL*-1368R | CTTTCCAAATTTCACAAGCAGCA | Reeves G, et al. [4] |
| *trnK*-2R | AACTAGTCGGATGGAGTAG | Mike T, et al. [1] |
| *matK*-19F | CGTTCTCATATTGCACTATG | Mike T, et al. [1] |
| *matK*-1867R | TTGCAGTTTTCATTGCACACG | Liu ZJ, et al. [5] |
| *matK*-147F | AACAAAACTTCCTATATCCGCT | Liu ZJ, et al. [5] |
| *matK*-1167R | CATTTGATTTCTTACTACC | Liu ZJ, et al. [5] |
| *matK*-1149F | GGTAGTAAGAAATCAAATG | Liu ZJ, et al. [5] |
| *matK*-969R | CTTTTCCTTGATATCGAACAT | Liu ZJ, et al. [5] |
| *matK*-731F | AAGAAAAGATTCTTTTGGTTCC | Liu ZJ, et al. [5] |
| *psaB*-NY159 | ACGCGTCGTATTTGGTTTGGTATTGC | Cameron KM, et al. [6] |
| *psaB-*NY160 | CAATGCCAATAAAAAGTAACCCATCC | Cameron KM, et al. [6] |

References

1. Mike T, Lena S, Joachim WK (1999) The phylogenetic relationships and evolution of the Canarian laurel forest endemic *Ixanthus viscosus* (Aiton) Griseb. (Gentianaceae): evidence from *matK* and ITS sequences, and floral morphology and anatomy. Plant Systematics and Evolution 218: 299–317.

2. Sulaiman SF, Culham A, Harborne JB (2003) Molecular phylogeny of Fabaceae based on *rbcL* sequence data: with special emphasis on the tribe Mimoseae (Mimosoideae). Asia Pacific Journal of Molecular Biology and Biotechnology 11: 9–35.

3. Zhai JW, Zhang GQ, Chen LJ, Xiao XJ, Liu KW, et al. (2013) A new orchid genus, *Danxiaorchis*, and phylogenetic analysis of the tribe Calypsoeae. PloS ONE 8: e60371.

4. Reeves G, Chase MW, Goldblatt P, Rudall P, Fay MF, et al. (2001) Molecular systematics of Iridaceae: evidence from four plastid DNA regions. American Journal of Botany 88: 2074–2087.

5. Liu ZJ, Chen LJ, Chen SC, Cai J, Tsai WC, et al. (2011) *Paraholcoglossum* and *Tsiorchis,* two new orchid genera established by molecular and morphological analyses of the *Holcoglossum* alliance. PLoS ONE 6 (10): e24864.

6. Cameron KM (2004) Utility of plastid *psaB* gene sequences for investigating intrafamilial relationships within Orchidaceae. Molecular Phylogenetics and Evolution 31: 1157-1180.
